# Microtubules Accelerate the Kinase Activity of Aurora-B by a Reduction in Dimensionality

**DOI:** 10.1371/journal.pone.0086786

**Published:** 2014-02-03

**Authors:** Michael Noujaim, Susanne Bechstedt, Michal Wieczorek, Gary J. Brouhard

**Affiliations:** Department of Biology, McGill University, Montréal, Québec, Canada; Institut de Génétique et Développement de Rennes, France

## Abstract

Aurora-B is the kinase subunit of the Chromosome Passenger Complex (CPC), a key regulator of mitotic progression that corrects improper kinetochore attachments and establishes the spindle midzone. Recent work has demonstrated that the CPC is a microtubule-associated protein complex and that microtubules are able to activate the CPC by contributing to Aurora-B auto-phosphorylation *in trans*. Aurora-B activation is thought to occur when the local concentration of Aurora-B is high, as occurs when Aurora-B is enriched at centromeres. It is not clear, however, whether distributed binding to large structures such as microtubules would increase the local concentration of Aurora-B. Here we show that microtubules accelerate the kinase activity of Aurora-B by a “reduction in dimensionality.” We find that microtubules increase the kinase activity of Aurora-B toward microtubule-associated substrates while reducing the phosphorylation levels of substrates not associated to microtubules. Using the single molecule assay for microtubule-associated proteins, we show that a minimal CPC construct binds to microtubules and diffuses in a one-dimensional (1D) random walk. The binding of Aurora-B to microtubules is salt-dependent and requires the C-terminal tails of tubulin, indicating that the interaction is electrostatic. We show that the rate of Aurora-B auto-activation is faster with increasing concentrations of microtubules. Finally, we demonstrate that microtubules lose their ability to stimulate Aurora-B when their C-terminal tails are removed by proteolysis. We propose a model in which microtubules act as scaffolds for the enzymatic activity of Aurora-B. The scaffolding activity of microtubules enables rapid Aurora-B activation and efficient phosphorylation of microtubule-associated substrates.

## Introduction

Aurora-B is a serine/threonine kinase that controls progression through each phase of mitosis, namely the construction of the mitotic spindle, the segregation of chromosomes, and the completion of cytokinesis. For example, Aurora-B ensures the proper attachment of chromosomes to the microtubules of the mitotic spindle through the correction of microtubules attached to the wrong kinetochore (merotelic attachments). The inability to correct these misattachments is the major source of chromosome instability in cancer cells, which is an underlying cause of aneuploidy. A central question is how Aurora-B activation is governed.

The first Aurora kinase, Ipl1, was identified in a screen for genes that cause an increase in ploidy in *S. cerevisiae* [1]. The family received its name from a *Drosophila* mutant that formed monopolar spindles reminiscent of the aurora borealis [2]. While fungi have one Aurora kinase (Ipl1 in *S. cerevisiae*, Ark1 in *S. pombe* [3]), metazoans have two kinases that regulate progress through mitosis: Auroras A and B. Aurora-B is part of a protein complex known as the chromosome passenger complex (CPC), so-named because it hitches a ride on chromosomes, which carry the complex to the spindle equator (reviewed in [4]). The core of the CPC is composed of Aurora-B, INCENP (INner CENtromere Protein, the first component identified [5]), Survivin [6], and Borealin (also known as Dasra) [7]. This functional module is conserved from yeast to humans. Alpha-helices of human INCENP, Survivin, and Borealin form a triple-helical bundle that links these CPC components into a single structural unit [8]. After chromosome condensation, the CPC is initially located at the centromere, where Aurora-B helps correct improper kinetochore attachments by phosphorylating several components of the kinetochore [9], [10], notably the Ndc80 complex [11] as well as centromeric MCAK [12], a microtubule depolymerase. Consistent with this, cells enter anaphase with improperly attached and misaligned chromosomes following impairment of Aurora-B function in XTC cells [13] and HeLa cells [14]. At the transition to anaphase, the CPC releases from chromosomes and binds to the spindle midzone. There, Aurora-B creates a gradient of phosphorylation centered on the midzone [15] that functions in spindle midzone organization and cytokinesis [16], [17].

Aurora-B becomes active through binding to activators and auto-phosphorylation in trans. Like all protein kinases, Aurora-B has a bilobed structure, including a hydrophobic pocket in the N-lobe that must be filled by a complementary hydrophobic motif in order for the kinase to be active [18]. Unlike other kinases, however, the complementary hydrophobic motif is not provided by C-terminal domains of Aurora-B itself. Rather, the complementary motif is provided by a C-terminal hydophobic region of INCENP, known as the INBox motif [19]. Aurora-B engages in auto-phosphorylation *in trans*, phosphorylating a crucial threonine on the activation loop that blocks the kinase active site (the T-loop). Human Aurora-B may also dimerize via a domain swap of the activation loop [20]. Further activation is achieved when Aurora-B phosphorylates a site on the INBox (the TSS-motif) [21]. The kinetics of auto-activation depend on the kinase concentration. Aurora-B is constitutively active when purified from *E. coli*, indicating that a high local concentration of Aurora-B is sufficient to stimulate activation. Similarly, linkage of *Xenopus* Aurora-B by binding to anti-INCENP antibodies also triggers activation [22].

Enrichment of Aurora-B on the inner centromere is thought to drive activation by increasing the local concentration of Aurora-B [22]. Centromere localization also plays a critical role in current models for sensing proper kinetochore attachments [23], [24]. Recent work, however, has implicated microtubules in Aurora-B activation; indeed, microtubules are required for the activation of Aurora-B *in vitro* [25]. We now know that the CPC binds to microtubules via INCENP. The *S. cerevisiae* homologue Sli5 [26] and human INCENP [27] both co-sediment with microtubules by centrifugation *in vitro* and co-localize with microtubules in the spindle midzone. The CPC binds to microtubules via INCENP's coiled-coil domain, and deletion of this domain results in a breakdown in spindle assembly in *Xenopus* egg extracts [28]. This result indicates that active Aurora-B contributes to microtubule polymerization, perhaps through inhibition of MCAK [12]. These interactions create a positive feedback loop, wherein active Aurora-B contributes to microtubule polymerization, which in turn contributes to Aurora-B activation [28]. Microtubule-based activation of Aurora-B was not sufficient for cell-cycle progression in *Xenopus* extracts, however, as deletion of the centromere-targeting motif of INCENP also lead to failures in spindle assembly [28]. In contrast, Campbell and Desai showed that the centromere localization of the CPC is not essential in budding yeast [29], suggesting that microtubule localization may be sufficient to enable auto-activation in some organisms. Based on these differing results, the relative importance of centromeres and microtubules in Aurora-B activation is an open question.

Centromeres are small structures, so it is easy to see how centromere localization would increase the local concentration of Aurora-B and contribute to kinase activation. In other words, centromeres “cluster” Aurora-B. What remains unclear is how very large structures, such as the microtubules of the mitotic spindle, can accelerate Aurora-B activation. Were Aurora-B to distribute throughout the microtubules of the spindle, the local concentration of Aurora-B may not, in fact, increase, because spreading Aurora-B over such large structures would not lead to clustering. We wanted to understand how microtubules contribute to Aurora-B activation and substrate targeting. Here we show that Aurora-B binds to microtubules and undergoes one-dimensional (1D) diffusion on the lattice. Microtubule binding accelerates Aurora-B activation and increases the phosphorylation of microtubule-associated substrates but decreases the phosphorlyation of substrates that do not bind microtubules. We propose that 1D diffusion on microtubules creates a “reduction in dimensionality” [30] that accelerates Aurora-B kinase activity. This mechanism is similar to the accelerated targeting of site-specific DNA-binding proteins to their sequences [31].

## Results

### Microtubules restrict Aurora-B activity to MAP substrates

**Figure 1:**
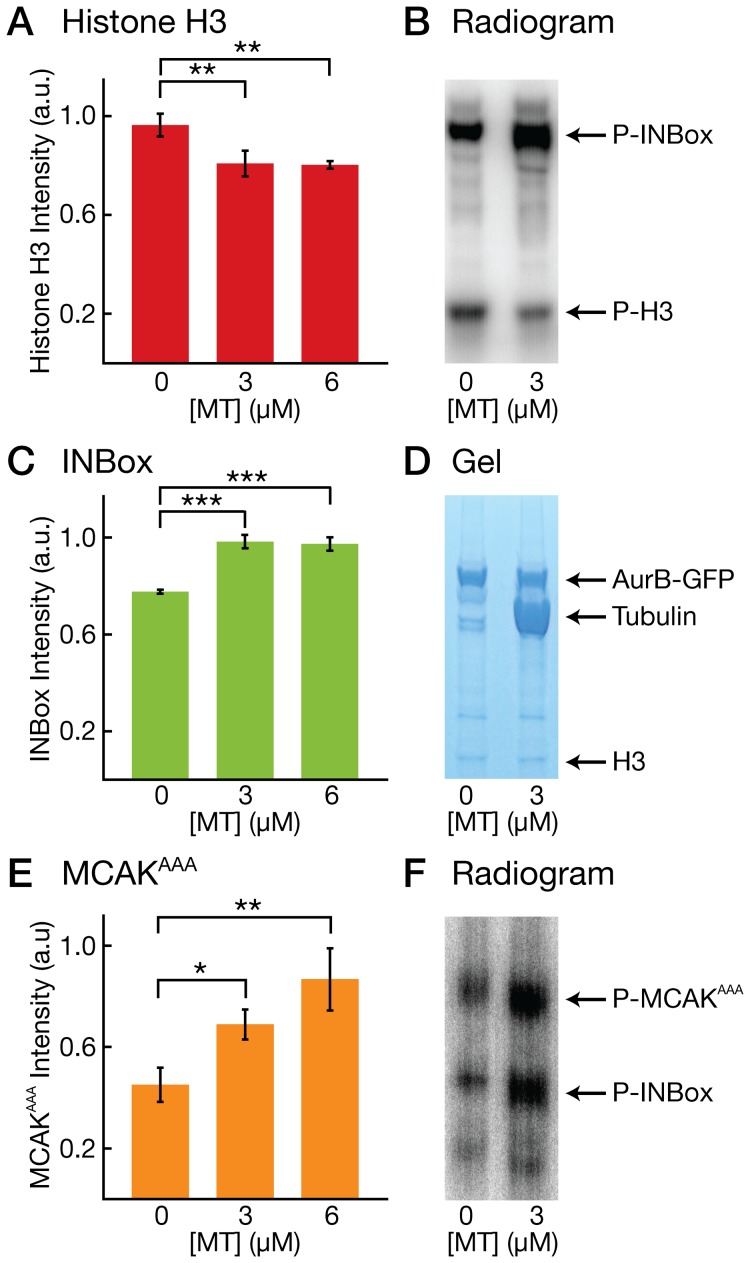
Microtubules sequester Aurora-B activity toward microtubule-associated substrates. (**A**) Bar graph showing the normalized Histone H3 band intensity in 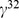
P radiograms at 3 different microtubule (MT) concentrations. (**B**) Image of a 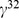
P radiogram showing radioactive bands corresponding to phosphorylated INBox motif (labeled) and Histone H3 (labeled) at 2 different microtubule concentrations. (**C**) Bar graph showing the normalized INBox band intensity in 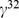
P radiograms at 3 different microtubule (MT) concentrations. (**D**) Image of an SDS-PAGE gel showing bands corresponding to Aurora-B-GFP (labeled), tubulin (labeled), and Histone H3 (labeled) at 2 different microtubule concentrations. The band for the INBox construct, at 52 kDa, is masked by the tubulin band. (**E**) Bar graph showing the normalized MCAK^AAA^ band intensity in 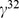
P radiograms at 3 different microtubule (MT) concentrations. (**F**) Image of a 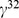
P radiogram showing radioactive bands corresponding to phosphorylated MCAK^AAA^ (labeled) and the INBox motif (labeled) at 2 different microtubule concentrations.

The CPC binds to microtubules via the coiled-coil domain of INCENP [28]. In order to reconstitute the interaction of the CPC with microtubules, we expressed and purified a minimal CPC complex consisting of the coiled-coil domain and INBox of INCENP (a.a. 491–873) and Aurora-B, which we have termed CCA (for coiled-coil Aurora-B), using a bicistronic expression vector. The cloning site in the expression vector contained an N-terminal 6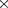-His tag and a C-terminal EGFP tag followed by a Strep-tag II [32]. The use of two purification tags allowed us to first pull on the INBox via the His tag and then pull on Aurora-B via the Strep-tag II. This purification strategy is designed to remove uncomplexed proteins. Our CCA-GFP purification yielded a highly-purified complex in which Aurora-B and the INBox motif are present in an approximately equimolar ratio (see Figure S1 in File S1). In order to test how microtubules affect the performance of the CCA, we set up *in vitro* kinase assays with 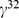
P-ATP. We started the reaction with 1 µM CCA, different concentrations of paclitaxel-stabilized microtubules (from 0 to 6 µM), and 2 µM Histone H3, a conventional Aurora-B substrate that has not been reported to interact with microtubules. After 30 minutes, we measured the intensity of bands in ^32^P-autoradiograms corresponding to phosphorylated Histone H3 using densitometric analysis. We observed that microtubules reduced the amount of phosphorylated Histone H3. Figure 1A shows a plot of the normalized Histone H3 band intensity at different microtubule concentrations. In the absence of microtubules, we measured a Histone H3 band intensity of 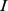 = 0.98
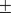
0.05 a.u., and this intensity was significantly reduced in the presence of microtubules (e.g., for 3 µM microtubules, 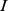 = 0.81
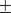
0.05 a.u., 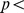
0.01, see Fig. 1B for example radiogram). In this experiment, as well as those described below, we also tested a CCA complex with a C-terminal Strep-tag II but without GFP. We observed no differences in the performance of the CCA complex without the GFP tag. There are two interpretations of this result. First, Histone H3 could bind to microtubules in a way that blocks access of Aurora-B to Histone H3. Although Histone H3 has not been reported to bind to microtubules, histones are basic proteins that may interact electrostatically with microtubules, which are acidic. Alternatively, the CCA could interact with microtubules in such a way that the microtubules sequester the kinase activity of Aurora-B away from substrates that do not interact with microtubules.

If microtubules sequester Aurora-B, it is possible that the phosphorylation of well-established microtubule-associated substrates will be enhanced. One such substrate is the CCA itself. To assess the effect of microtubles on CCA auto-phosphorylation, we quantified the ^32^P-autoradiogram bands corresponding to INCENP's INbox motif in the experiment above. Although Aurora-B typically phosphorylates itself in trans prior to purification from *E. coli*, we observed additional auto-phosphorylation in our *in vitro* kinase assays. As predicted, the level of CCA auto-phosphorylation increased in the presence of microtubules. Figure 1C shows a plot of the normalized band intensity of the INBox motif at different microtubule concentrations. In the absence of microtubules, we measured an INBox band intensity of 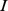 = 0.77
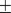
0.008 a.u., and this intensity was significantly increased in the presence of microtubules (e.g., for 3 µM microtubules, 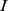 = 0.98
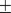
0.03 a.u., 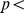
0.001, see also Fig. 1B). Figure 1D shows an SDS-PAGE gel demonstrating that protein levels remained constant across experiments. These results indicate that microtubules increase the kinase activity of Aurora-B toward microtubule-associated substrates.

In order to determine whether this phenomenon was general or specific to the auto-phosphorylation reaction, we tested a second microtubule-associated substrate, namely the microtubule depolymerase MCAK, a prominent Aurora-B target [12]. Adding MCAK to an *in vitro* kinase assay with microtubules is tricky, however, because MCAK will depolymerize the microtubules. To prevent MCAK from doing so, we expressed and purified an MCAK construct in which three amino acids essential for depolymerization, the KVD finger, are substituted with alanines (MCAK-KVD
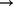
AAA, hereafter MCAK^AAA^ [33]). We measured the extent of MCAK^AAA^ phosphorylation by Aurora-B using different concentrations of microtubules, and we observed the microtubules increased the level of MCAK^AAA^ phosphorylation (Fig. 1E). In the absence of microtubules, we measured an MCAK^AAA^ band intensity of 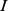 = 0.45
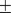
0.07 a.u., and this intensity was significantly increased in the presence of microtubules (e.g., for 3 µM microtubules, 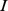 = 0.7
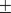
0.06 a.u., 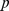 = 0.01, see Fig. 1F for example radiogram). We conclude that microtubules increase the kinase activity of Aurora-B against multiple microtubule-associated substrates, and that microtubules likely play a general role in enhancing Aurora-B activity. We cannot exclude, however, the possibility that the Histone H3 result (Fig. 1A) is explained by the binding of Histone H3 to microtubules in a way that blocks kinase access.

### Aurora-B diffuses on microtubules in a 1D random walk

**Figure 2:**
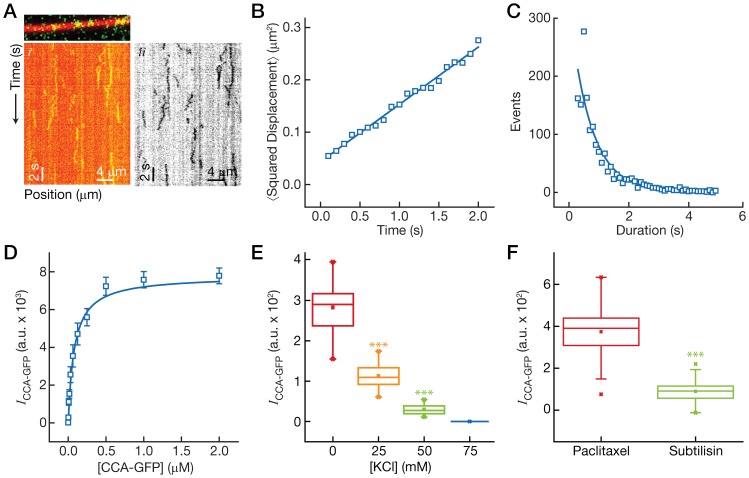
The CCA diffuses on microtubules via electrostatic interactions. (**A**) Still image (top) and kymographs (bottom) of the CCA-GFP (green) interacting with microtubules (red). The back-and-forth movements of a 1D random walk are evident. (***i***) A color-combined kymograph of CCA-GFP diffusion. (***ii***) An inverted grayscale image of the CCA-GFP signal shown in (*i*). (**B**) Plot of the mean squared displacement, 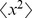 against time for the CCA-GFP. A linear relationship is observed, and slope of the fit (blue line), 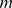, is related to the diffusion coefficient by 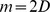. (**C**) Histogram of the duration of CCA-GFP binding events. An exponential decay fit (blue line) gives a mean duration of 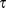 = 1.5 s. (**D**) Plot of the intensity of the CCA-GFP on the microtubule lattice against the concentration of the CCA-GFP. The data is well described by a conventional binding isotherm (blue line). (**E**) Box plot of the intensity of the CCA-GFP on microtubules at four different salt concentrations. (**F**) Box plot of the intensity of the CCA-GFP on microtubules for paclitaxel-stabilized microtubules and subtilisin-digested microtubules (labeled).

We considered two possible mechanisms by which microtubules could enhance the kinase activity of Aurora-B toward microtubule-associated substrates. First, microtubules could induce a conformational change in Aurora-B that enhances its catalytic activity. Alternatively, microtubules could increase the rate at which Aurora-B encounters its substrates. In order to distinguish between these hypotheses, we chose to visualize the interactions of our CCA construct with microtubules using the single molecule assay for microtubule-associated proteins [34]. We expressed and purified a GFP-tagged CCA construct and introduced this protein into a microscope flow chamber with surface-immobilized, rhodamine-labeled microtubules. We observed the CCA binding to the MT lattice and diffusing along it in a one-dimensional random walk. Figure 2A shows a kymograph of this “lattice diffusion,” which is a common mode of interaction for microtubule-associated proteins [35]. We tracked the motion of 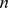 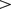
500 CCA-GFP molecules using in-house tracking software. Figure 2B shows a plot of the mean-squared displacement of the CCA-GFP against time. We fit the data to a line; the slope of this line gave us a diffusion coefficient of 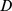 = 0.055
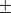
0.001 µm^2^ s^−1^. We also measured the lifetimes of the CCA-GFP trajectories (see Figure 2C). The lifetimes were distributed exponentially, with a mean residence time on the lattice of 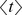 = 1.5
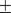
0.02 s, after correcting for photobleaching, from which we can infer a dissociation rate constant of 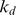 = 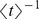 = 0.66 s^−1^.

In order to measure the affinity of the CCA-GFP for microtubules, we measured the fluorescence intensity on the microtubule lattice at increasing CCA-GFP concentrations. Figure 2D shows a plot of the GFP intensity against concentration, which was well-described by a conventional binding isotherm. The fit produced an equilibrium dissociation constant of 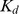 = 0.08 µM. From the 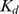 and the 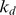, we can infer an association rate constant of 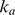 = 8.25 µM^−1^ s^−1^, which is consistent with the CCA binding to microtubules in a diffusion-limited reaction. These results indicate that the CCA diffuses on the microtubule lattice and that the parameters describing this diffusion are similar to those reported for other microtubule-associated proteins.

Most if not all of the microtubule-associated proteins that diffuse on microtubules do so via electostatic interactions with the microtubule lattice, e.g., Kif1A [36], MCAK [37], and the Ndc80 complex [38]. INCENP's coiled-coil domain is enriched in positively charged amino acids and may interact with the negatively-charged C-terminal tails of tubulin [28]. In order to test whether the CCA-GFP diffuses on microtubules by electrostatic interactions, we introduced CCA-GFP molecules to surface-immobilized microtubules in the presence of increasing concentrations of salt. We observed that salt significantly attenuated CCA-GFP binding. Figure 2E shows a plot of the CCA-GFP intensity on microtubules against salt concentration. In the absence of added salt, we measured a CCA-GFP signal of 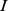 = 277
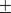
65 a.u. This signal was reduced by the addition of 25 mM KCl (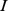 = 117
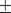
35 a.u., 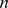 = 50, 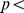
0.001), reduced further by the addition of 50 mM KCl (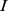 = 27
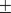
15 a.u., 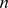 = 50), and abolished by further addition of salt. In order to confirm that the CCA-microtubule interactions required the C-terminal tails of tubulin, we removed these tails by proteolytic cleavage with subtilisin. We introduced CCA-GFP molecules into a flow chamber containing both brightly-labeled microtubules and dimly-labeled, subtilisin-digested microtubules. We observed that the GFP signal on subtilisin-digested microtubules were significantly dimmer (see Fig. 2F, 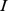 = 375
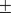
111 a.u. vs. 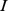 = 89
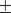
47 a.u., 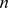 = 80, 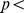
0.001). These results indicate that CCA-microtubule interactions are electrostatic and involve the C-terminal tails of tubulin. Similar results relating to the electrostatic interaction of the CPC with microtubules were recently reported for the budding yeast CPC [39].

### Microtubules accelerate Aurora-B auto-activation by a “reduction in dimensionality”

**Figure 3:**
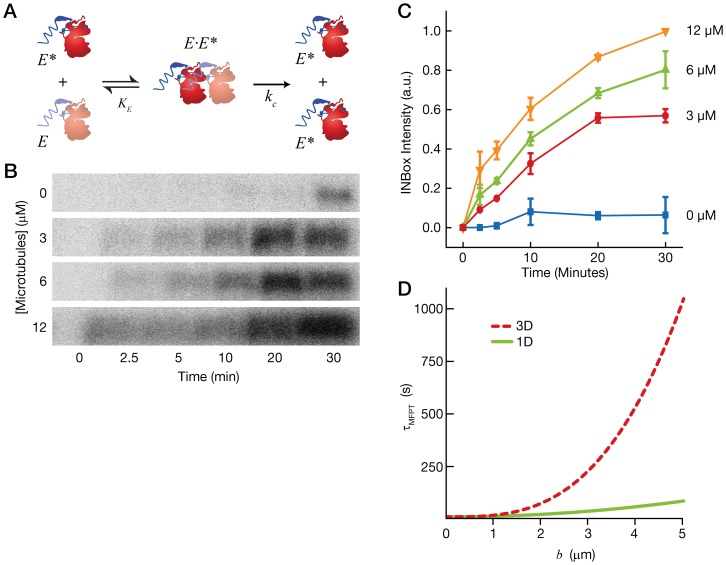
Microtubules accelerate Aurora-B activation by a reduction in dimensionality. (**A**) Schematic of a model for Aurora-B auto-activation. An active kinase (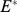, bright red) binds to an inactive kinase (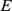, dim red) with an equilibrium constant, 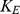, to form a complex, 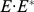. The kinase reaction occurs with a catalytic constant, 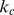, producing two active kinases (*right*). (**B**) Images of 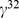
P radiograms showing radioactive bands corresponding to phosphorylated INBox motif. The images correspond to 4 microtubule concentrations (labeled at *left*). The lanes in the radiogram correspond to different time points (labeled at *bottom*). At higher microtubule concentrations, the radioactive bands become more intense at earlier time points. (**C**) Plot of the INBox motif band intensity against time for 4 microtubule concentrations. (**D**) Plot of the mean first passage time, 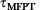, against the size of the confining space in which the molecules diffuse, 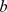. The plot shows both 3D diffusion (red hashed line) and 1D diffusion (green solid line) using the diffusion coefficients for Aurora-B (3D case, estimated; 1D case, measured). 1D diffusion is significantly faster when the molecules diffuse within spaces 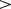
2 µm in radius.

In order to understand the role of diffusion in Aurora-B kinase activity, we adopted a simple kinetic model that describes Aurora-B auto-activation. In the model, which is based on the work of Wang and Wu [40] and drawn in Figure 3A, we assume that small amounts of active kinase (labeled 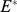) bind to inactive kinases (labeled 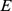) with an equilibrium dissociation constant, 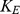. The active kinase then phosphorylates the inactive kinase with a catalytic rate constant, 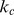, producing an additional active kinase, 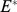. The Supplemental Information (File S1) contains a derivation of the amount of active kinase that accumulates as a function of time. As a caveat, we note that our model considers kinase activation as a single-step process, when in fact Aurora-B activation is a multi-step process comprised of binding to INCENP and phosphorylation *in trans* on multiple residues. Nevertheless, a prediction of our model is that decreasing the value of 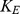 leads to a faster accumulation of active kinase. Our hypothesis is that microtubules change the equilibrium dissociation constant, 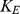, by increasing the rate of association of the active and inactive kinases.

We tested the prediction of our model by measuring the amount of active kinase that accumulates over time in the presence of increasing concentrations of microtubules. We began the experiment by treating our purified CCA construct with 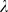-phosphatase to partially inactivate the population of kinases, which are otherwise constitutively active due to their expression in *E. coli*. Next, we inhibited 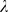-phosphatase with sodium orthovanadate and started the kinase reaction. The band intensity of the INBox motif on ^32^P-autoradiograms was measured at six intervals over a 30-minute time course. We observed negligible auto-activation in the absence of microtubules. In contrast, microtubules stimulated auto-activation, and active kinase accumulated faster at higher microtubule concentrations. Figure 3B shows a plot of the INBox motif band intensity as a function of time at 4 different microtubule concentrations. In the absence of microtubules, we observed negligible kinase activity due to the inactivation of the kinase with 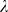-phosphatase. At every time point, the level of INBox phosphorylation is higher at increasing microtubule concentration. These results indicate that microtubules accelerate the auto-activation of the CCA complex, which, in our model, implies that microtubules increase the rate of association of active and inactive kinases.

How do microtubules increase the rate of association of Aurora-B in trans? One hypothesis is that the diffusion of Aurora-B on microtubules creates a “reduction in dimensionality,” wherein the kinases search for one another by 1D diffusion on the microtubule lattice rather than 3D diffusion is solution [30]. While intuitive, “reduction in dimensionality” is not always effective and depends critically on the ratio of the diffusion coefficients in 3D and in 1D [41]. To test whether “reduction in dimensionality” accelerates the rate of association in our case, we calculated the mean first-passage time for two proteins diffusing in 3D solution, and we compared this to the mean first passage time for two proteins diffusing in 1D on the microtubule lattice. For the 3D case, we used the predicted diffusion coefficient of a globular protein of 100 kDa, whereas for the 1D case, we used the diffusion coefficient for the CCA-GFP on microtubules measured above. Our model assumes the proteins are confined within a space of known size (e.g., within a cell). Figure 3D shows a plot of the mean first passage times as a function of the size of the confining space. Our model shows that, for molecules confined within spaces of 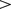
2 µm in radius, 1D diffusion confers a 6-fold advantage in mean first passage time, an advantage that grows exponentially as the confining space becomes larger. The mean first passage time may be further reduced if the CCA complexes undergo interspersed periods of 3D and 1D diffusion, as occurs in most models for the interaction of site-specific DNA-binding proteins with their target sequences [31].

### Disrupting microtubule binding releases Aurora-B to soluble substrates

**Figure 4:**
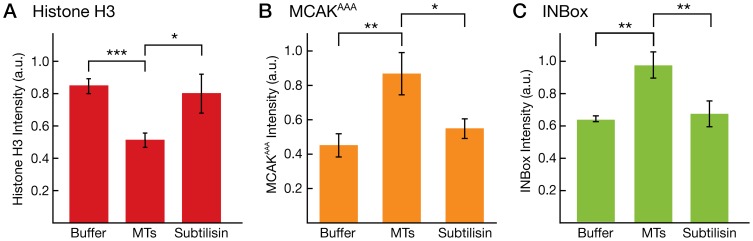
Disruption of Microtubule binding releases Aurora-B to soluble substrates. (**A**) Bar graph showing the normalized Histone H3 band intensity in 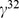
P radiograms under three conditions: no microtubules (Buffer), paclitaxel-stabilized microtubules (MTs), and subtilisin-digested microtubule (Subtilisin). (**B**) Bar graph showing the normalized MCAK^AAA^ band intensity in 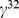
P radiograms under three conditions: no microtubules (Buffer), paclitaxel-stabilized microtubules (MTs), and subtilisin-digested microtubule (Subtilisin). (**C**) Bar graph showing the normalized INBox motif band intensity in 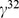
P radiograms under three conditions: no microtubules (Buffer), paclitaxel-stabilized microtubules (MTs), and subtilisin-digested microtubule (Subtilisin). Note that the Histone H3, MCAK^AAA^, and INBox band intensities were normalized independently, so the data cannot be used to compare the absolute levels of phosphorylation across the different substrates.

If microtubule binding confers an advantage to Aurora-B in terms of auto-activation and targeting of microtubule-associated substrates, then disrupting microtubule binding should reverse this advantage. To test this prediction, we asked whether subtilisin-digested microtubules, on which the CCA complex does not diffuse, confer the same advantage as normal microtubules, and we asked how subtilisin-digested microtubules compare to the microtubule-free control. We started *in vitro* kinase reactions with our CCA complex using Histone H3, MCAK^AAA^, and the INBox motif as substrates. We measured the band intensity for these substrates on ^32^P-autoradiograms under conditions without microtubules, with 3 µM microtubules, or with 3 µM subtilisin-digested microtubules. Figure 4A shows a plot of normalized Histone H3 band intensities in these three conditions. Consistent with our previous observations, Histone H3 band intensity was increased in the absence of microtubules (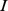 = 0.94
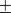
0.05 a.u. vs. 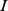 = 0.57
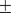
0.05 a.u., 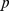 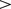
0.001). Notably, subtilisin-digestion of the microtubules restored the Histone H3 band intensities to the level observed in the absence of microtubules (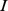 = 0.90
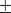
0.13 a.u., 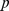 = 0.6). There are two interpretations of this result. First, subtilisin-digestion could relieve the sequestration of the CCA complex onto microtubules, thereby releasing the CCA to phosphorylate soluble substrates. Alternatively, Histone H3 could interact with the C-terminal tails of tubulin in such a way as to block its phosphorylation; subtilisin-digestion could relieve this block. We then tested our well-established microtubule-associated protein substrates. MCAK^AAA^ band intensities, shown in Figure 4B and normalized independently, were increased in the presence of microtubules, (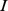 = 0.87
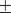
0.12 a.u. vs. 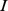 = 0.45
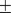
0.07 a.u., 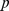 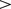
0.01), as observed previously, while subtilisin-digested microtubules had a reduced band intensity (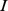 = 0.55
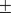
0.06, 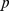 = 0.13 when compared to buffer control). Figure 4C shows the results for the INBox motif. The normalized band intensities of the INBox motif were also increased in the presence of microtubules (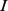 = 0.92
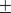
0.07 a.u. vs. 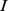 = 0.62
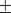
0.015 a.u., 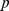 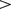
0.01), consistent with our previous observations. As expected based on the CCA's electrostatic interaction with microtubules, INBox motif band intensities were reduced in the presence of subtilisin-digested microtubules (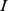 = 0.64
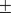
0.07 a.u., 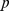 = 0.55 when compared to buffer control). These results indicate that subtilisin-digested microtubules do not confer an advantage to the CCA complex and that the C-terminal tails of tubulin are essential.

## Discussion

Due to its stiffness and resistance to compressive load, the microtubule cytoskeleton acts as a mechanical scaffold for cell division in the form of the mitotic spindle. But a different type of scaffold also exists in cells, namely the scaffolds that underlie a wide range of signal transduction pathways [42]. An example is the structural protein Ste5 in *S. cerevisiae*. Ste5 brings the three core kinases of the MAP kinase mating pathway into close proximity with their downstream targets, thereby modulating the response to mating pheromone [43]. A scaffolding mechanism is also at work with membrane tethering of enzymes and proteins, such as the epidermal growth factor receptor (EGFR), a transmembrane tyrosine kinase that is activated by ligand-induced dimerization [44]. The intracellular kinase domains of EGFR alone are monomeric and ineffective at auto-phosphorylation in trans, even at high concentration [45]. Only when tethered to the membrane are the kinase domains able to effectively engage in the first step of signal transduction. We can consider Ste5 and the plasma membrane as “enzyme scaffolds:” a structure that links two or more enzymes or proteins so as to accelerate, promote, modulate, or enable a reaction cascade. The linkage can be rigid (Ste kinases) or fluid (EGFR kinase domain). The linkage can modulate a reaction cascade, as with the response to mating pheromone, or it can be a prerequisite for the reaction to occur, as with EGFR auto-activation.

Our results indicate that microtubules serve as enzyme scaffolds for Aurora-B by a mechanism analogous to membrane tethering. Aurora-B diffuses on the surface of microtubules via transient electrostatic interactions with the C-terminal tails of tubulin. The transition from 3D diffusion in solution to 1D diffusion on the microtubule corresponds to a “reduction in dimensionality” [30], and the result is accelerated reaction kinetics for Aurora-B activation and substrate targeting. A similar mechanism explains how MCAK rapidly targets microtubule ends [37] and how site-specific DNA-binding proteins target their sequences [31]. We cannot exclude the possibility that microtubules also induce a conformational change in Aurora-B, e.g., by bringing the N- and C-terminal domains into register [19], as proposed [25]. In addition, lattice diffusion may correctly orient proteins such as Aurora-B for productive intermolecular contact [46] in addition to increasing the rate of association of Aurora-B with itself and with its substrates. These models are not mutually exclusive, and our work demonstrates that, as with membrane-tethered receptor proteins, diffusion of two reaction partners on the surface of microtubules will give rise to accelerated reaction kinetics.

The diffusion of Aurora-B on microtubules may contribute to the gradient of kinase activity in the vicinity of kinetochores [24], which has been explained primarily in terms of the dynamics of Aurora-B at centromeres [47]. Aurora-B activity spreads away from the chromosome following centromere-activation [47], and diffusion on kinetochore microtubules may shape the profile and kinetics of the spatial gradient in kinase activity. Likewise, lattice diffusion may contribute to the gradient in Aurora-B activity observed at the spindle midzone [15]. The affinity of the CPC for microtubules is regulated by phosphorylation [48], and phosphorylation may also influence the diffusion coefficient of the CPC. The spatial gradients of Aurora-B activity are a unique reaction-diffusion system, in which centromere dynamics, microtubule lattice diffusion, and phosphorylation reactions give rise to subtle variations in kinase activity.

## Methods

### Cloning, expression, and protein purification

The coding sequence for INCENP a.a. 491–873 and Aurora B (the CCA complex) was PCR amplified using PfuX7 polymerase [49] from a bicistronic pET28 vector containing the coding sequences of both full-length *Xenopus laevis* INCENP and Aurora B (a gift from Dr. P. Todd Stukenberg). The amplified sequence, which preserved the cistron for Aurora-B, was cloned into a pHAT protein expression vector using the USER cloning method as described [50]. The pHAT vector contained an N-terminal 6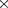-His tag and a C-terminal EGFP tag followed by a Strep-tag II [32]. Alternatively, the same sequence was cloned into a similar pHAT vector without a C-Terminal EGFP tag. Plasmids were transformed into the BL21(DE3) strain of *E. coli* at OD(600) = 0.4
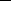
0.5 with 0.5 mM IPTG for 16 hours at 18°C. Cells were harvested by centrifugation, resuspended in 20 mM Tris (pH 7.9), 500 mM NaCl, and 10 mM imidazole and lysed using a French press in the presence of a protease inhibitor cocktail. The lysate was clarified by centrifugation at 100,000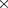 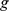 for 45 min at 4°C. The supernatant was loaded onto a 1 ml Ni^2+^-NTA affinity column (HisTrap SP HP, GE Healthcare), washed with 10
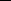
15 column volumes of 40 mM imidazole in Buffer B (50 mM Na_2_HPO_4_, pH 7.5, 300 mM NaCl, 10% glycerol) and eluted with 200 mM imidazole in Buffer B using a step gradient on an Akta Purifier FPLC system (GE Healthcare). Peak fractions were loaded onto a 0.5 ml Strep-Tactin Superflow gravity flow column (IBA, Germany), which bound the C-terminal Strep-tag II. Purified CCA complexes were eluted with 2.5 mM desthiobiotin in storage buffer (100 mM Tris/HCl, pH 8.0, 150 mM NaCl, 1 mM EDTA). In some cases, the Strep-tactin elution fractions were loaded onto a Superdex 200 size-exclusion column in order to verify that the proteins eluted as a single complex.

The pFastBac plasmid containing MCAK(KVD
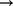
AAA), or MCAK^AAA^, was a gift of Dr. Claire Friel. MCAK^AAA^ was expressed in *Sf*9 insect cells (Invitrogen, USA) according to manufacturer's protocols. Cells were harvested by centrifugation, resuspended in 4 ml/g of lysis buffer (50 mM Na_2_HPO_4_, pH 7.5, 300 mM NaCl, 10 mM imidazole, 1 mM MgCl_2_, 10 µM ATP, 10% glycerol), and lysed by dounce homogenization. Lysates were clarified by centrifugation as described above. The supernatant was incubated in 1 ml His60 Ni^2+^ Superflow resin (Clontech, USA) on a shaker for 90 min at 4°C. The resin was washed 3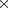 with lysis buffer and purified MCAK^AAA^ was eluted with 300 mM imidazole in lysis buffer.

Freshly-purified protein samples were aliquoted and flash-frozen in LN_2_ in the presence of 10% glycerol and stored in their respective elution buffers at 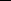
80°C. The purity of the samples was determined using SDS-PAGE and Coomassie Blue gel staining. Protein concentration was measured using a Bradford assay and absorbance at 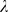 = 280 nm and 488 nm using a NanoDrop spectrophotometer (Thermo Scientific).

### Tubulin Preparations and Microtubule Polymerization

Tubulin was purified from juvenile bovine brains using a modified version of the Castoldi and Popov method [51], wherein the first polymerization cycle was performed in 100 mM PIPES instead of 1 M PIPES. Bovine brains were purchased from a commercial slaughterhouse (Abattoir Jacques Forget, Terrebone, Québec), which received cows from local farmers in neighboring regions of Québec. Animal husbandry and humane slaughter practice were carried out in accordance with Québec law. Labeling of tubulin with tetramethylrhodamine (TAMRA) was performed as described [52]; fluorescently-labeled tubulin was typically used at a labeling ratio of 1∶4 labeled∶unlabeled tubulin dimers. Tubulin aliquots were stored at a final concentration of 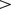
300 µM in BRB80 (80 mM PIPES, 1 mM MgCl_2_, 1 mM EGTA, pH 6.9), flash-frozen in LN_2_, and stored at 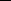
80°C. Tubulin was polymerized into microtubules as follows: a polymerization mixture was prepared with BRB80+32 µM tubulin+1 mM GTP+4 mM MgCl_2_+5% DMSO. The mixture was incubated on ice for 5 min, followed by incubation at 37°C for 30 min. The polymerized microtubules were diluted into pre-warmed BRB80+10 µM paclitaxel, centrifuged for 5 min at maximum speed in a Beckman Airfuge, and resuspended in BRB80+10 µM paclitaxel. Subtilisin-digested microtubules were prepared as follows: 40 µg/ml subtilisin was added to paclitaxel-stabilized microtubules and the mixture was incubated at 37°C for 1 hour. The digestion reaction was stopped by the addition of 2 mM PMSF. The subtilisin-digested microtubules were loaded onto a cushion of 60% glycerol in BRB80+10 µM paclitaxel and centrifuged for 5 min at maximum speed in a Beckman Airfuge to separate the C-terminal tails from the microtubules. The material on the cushion was carefully removed and the cushion was washed 2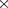 with BRB80. The pelleted microtubules were resuspended in BRB80+10 µM paclitaxel. The removal of the C-terminal tails was verified by Western Blot using a Tub 2.1 antibody (Sigma-Aldrich), which specifically recognizes the tails.

### *In vitro* Kinase Assays

*In vitro* kinase assays with the CCA complex were performed similarly to published protocols [25]. In brief, kinase reactions were initiated using 23 pmol recombinant CCA complex, 100 µM ATP, 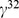
P-ATP (10 µCi), and 50 pmol of substrate (Histone H3 or MCAK^AAA^, as specified) in BRB80 buffer. Microtubules were added to the reaction by dilution from a stock solution; in these cases, paclitaxel was added to the reaction to a final concentration of 10 µM to maintain the stability of the microtubules. Paclitaxel was also added to the microtubule-free experiments for consistency across reactions. The reactions were performed at room temperature in a total reaction volume of 25 µl. For some experiments, the CCA complex was untreated after purification from *E. coli*. In other experiments, the CCA complex was treated with 400 units of 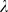-phosphatase for 1 hour at 30°C in order to remove activating phosphorylations accumulated in *E. coli*. The 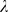-phosphatase was then inhibited by 1 mM sodium-orthovanadate (Na_3_VO_4_). Assay time points were quenched with SDS-sample buffer, analyzed by SDS-PAGE and phosphor-imaging. Briefly, gels were dried and exposed for 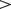
2 hours to Storage Phosphor screens. The screens were scanned using a GE Typhoon Trio+ phosphor-imager. Densitometric analysis was carried out in ImageJ to quantify phosphorylation. A rectangle was drawn over the first lane of the imaged gel and replicated over each subsequent lane. The integrated intensity of each band was measured using the gel analysis tools in ImageJ. Following background subtraction, all measured values were normalized to the highest observed intensity for the protein being assessed (e.g. phosphorylated Histone H3 band intensities were normalized to the highest measured Histone H3 band intensity in a given experiment). The normalization procedure simplifies the comparison of results from different substrates and different conditions, because the absolute values of the band intensities are a function of the intrinsic activity of the kinase towards a substrate, the exposure time, the quality of the Storage Phosphor screens, etc. Standard deviations were calculated from a minimum of 3 independent trials, and 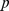-values were calculated using Welch's t-test. We note, however, that the same results and 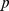-values within an experiment are obtained when raw intensity values are used.

### Total-Internal Reflection Fluorescence Microscopy

The single-molecule assay for kinesins and microtubule-associated proteins was performed as described [34], with specific modifications described below. The microscope setup uses a Zeiss Axiovert Z1 microscope chassis, 100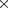 1.45 NA Plan-apo-chromat objective lens, and the Zeiss TIRF III slider. Diode-pumped solid-state lasers (Cobolt Jive, Cobolt Calypso) were coupled to fiber-optic cables in free space and introduced into the Zeiss slider. Epifluorescence used a PhotofluorII excitation source (89North) with wavelength-specific filter cubes (Chroma). Images were recorded using an Andor iXon+ DV-897 EMCCD camera. Microscope chambers were constructed using custom-machined mounts diagrammed in Gell et al. (2010) [34]. In brief, microscope cover glass were silanized as described [37]. A 22
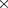
22 mm glass and an 18
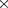
18 mm glass were separated by double-sided tape, such that a narrow channel was created for the exchange of solution. Fluorescently-labeled microtubules were adhered to silanized glass slides as described [53] using anti-rhodamine antibodies. The surrounding surface was passivated with blocking co-polymers to prevent non-specific protein binding. GFP-tagged CCA complexes were introduced into the reaction chamber and images were acquired using Metamorph (Molecular Devices). Simple measurements of microtubule intensities were made using the Linescan feature in Metamorph, and all intensity values are reported as mean 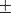 SD. The absolute values of intensity are a function of laser power, laser alignment, and camera gain. Camera gain settings and laser powers were chosen to maximize the sensitivity and dynamic range of the camera, which prevents direct comparisons in intensity values between experiments in some cases.

In order to measure the affinity of the CCA-GFP for microtubules, fluorescently-labeled microtubules were adhered to a cover glass surface in a flow chamber as described above. CCA-GFP molecules were introduced into the flow chamber at increasing concentrations and allowed to equilibrate. Images were recorded using Metamorph. The intensity of the CCA-GFP on microtubules was measured using the line-scan function in Metamorph. Data-points were fit to a Michaelis-Menten curve in Origin 8.5 to determine the equilibrium dissociation constant.

Single-molecule particle tracking was performed as described [37] using the Kalaimoscope software package (Transinsight GmbH, Dresden, Germany). In brief, single molecules were identified as diffraction-limited point-spread-functions. The pixel-intensity pattern was fit to a 2D Lorentzian function to provide the (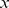,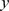) coordinates of the signal. Signals from consecutive frames were linked into trajectories using a cost-minimization function. The data for these trajectories was exported to MATLAB (MathWorks, Natick, MA, USA) for further analysis. The data were fit to an exponential decay function using OriginPro 8.5. Corrections for photobleaching were performed as described [46]. All experiments reported were repeated in a minimum of three independent trials.

## Supporting Information

File S1 **The Supplemental Information includes Figure S1, an SDS-PAGE gel of a CCA-GFP purification, a mathematical model for CCA auto-activation, and a calculation of the mean-first-passage-time for 3D and 1D diffusion of the CCA.**
(PDF)
