# Supplementary material for: Microtubules Accelerate the Kinase Activity of Aurora-B by a Reduction in Dimensionality
Source: PLoS One. 2014 Feb 3;9(2):e86786. doi: 10.1371/journal.pone.0086786 (PMC3912212; doi:10.1371/journal.pone.0086786)
Supplement: File S1 — The Supplemental Information includes Figure S1, an SDS-PAGE gel of a CCA-GFP purification, a mathematical model for CCA auto-activation, and a calculation of the mean-first-passage-time for 3D and 1D diffusion of the CCA. (PDF) [file pone.0086786.s001.pdf]

# Microtubules accelerate the kinase activity of Aurora-B by a reduction in dimensionality

Michael Noujaim[1], Susanne Bechstedt[1], Michal Wieczorek[1], Gary J. Brouhard[1,*]
**1 Department of Biology, McGill University, Montréal, Québec, Canada**
**∗ E-mail: gary.brouhard@mcgill.ca**

### Supplemental Information

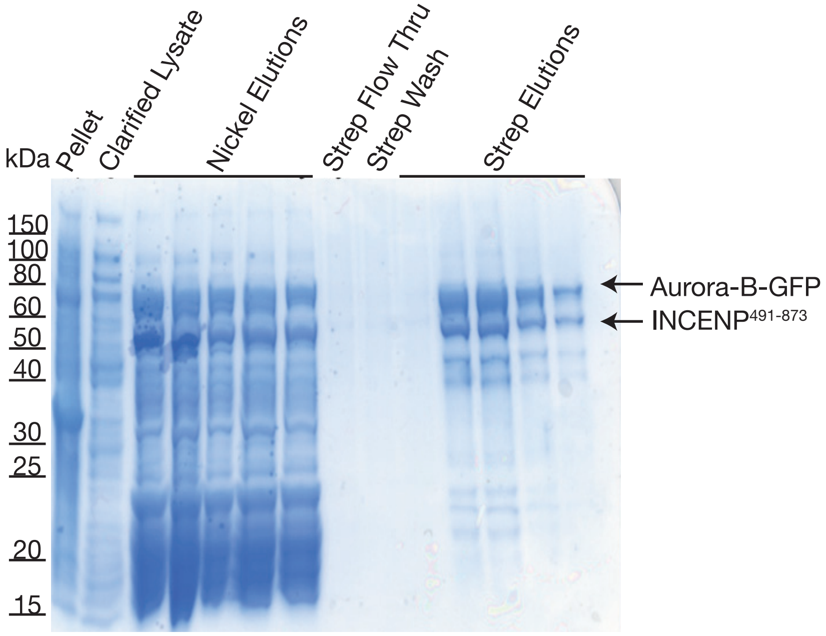


**Figure S1. Purification of CCA-GFP.** An SDS-PAGE gel from a purification of the CCA-GFP. The two proteins in the complex are initially visible in the elutions from the Ni-NTA column (Nickel Elutions, labeled). The complex is further purified using the Strep-tactin column (Strep Elutions, labeled).

### CCA Auto-activation Model

Following the formulation of Wang and Wu [1], an intermolecular autophosphorylation reaction scheme can be shown as:

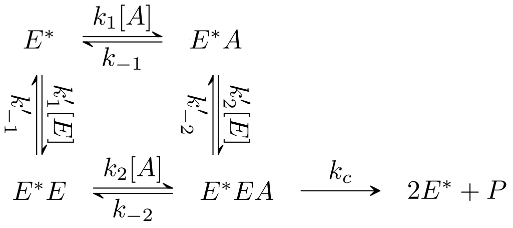

where $E$, $E^*$, $A$, and $P$ are unphosphorylated kinase, phosphorylated kinase, ATP and ADP, respectively. Equilibrium dissociation constants are defined as:

$$K'_A = \frac{[E^*][A]}{[E^*A]} = \frac{k_{-1}}{k_1}$$

$$K'_E = \frac{[E^*][E]}{[E^*E]} = \frac{k'_{-1}}{k'_1}$$

$$K_A = \frac{[E^*E][A]}{[E^*EA]} = \frac{k_{-2}}{k_2}$$

$$K_E = \frac{[E^*A][E]}{[E^*EA]} = \frac{k'_{-2}}{k'_2}$$

It should be noted that $K_A K'_E = K'_A K_E$. The Michaelis-Menten constant, $K_M$ and turnover rate, $k_{cat}$, are:

$$K_M = K_E \frac{(K'_A + [A])}{(K_A + [A])} \tag{1}$$

and

$$k_{cat} = \frac{k_c[A]}{K_A + [A]} \tag{2}$$

Certain assumptions simplify Equations 1 and 2. First, we assume that $E^*$ and $E^*A$ bind to $E$ with the same affinity, which is to say that ATP does not change the affinity. Then $K_E = K'_E$, which means that $K_A = K'_A$ and therefore Equation 1 reduces to $K_M = K_E$. Second, we assume that $[A]$ is sufficiently high relative to all other species (at least 100 $\mu$M used in experiments) so that Equation 2 reduces to $k_{cat} = k_c$.

To solve the scheme, let the total concentration of kinase be $[T]_0 = [E]_0 + [E^*]_0 = [E] + [E^*] + [E^*A] + 2[E^*E] + 2[E^*EA]$, where $[E]_0$ and $[E^*]_0$ are the initial concentrations of unphosphorylated and phosphorylated kinase, respectively. The above simplifications let us write the impicit integrated rate equation found in [1] as:

$$t = \frac{-1}{k_c}\left(\ln\frac{x}{x_0} + \frac{K_E + [T]_0}{[T]_0}\ln\frac{(x - K_E - 2[T]_0)(x_0 - K_E)}{(x_0 - K_E - 2[T]_0)(x - K_E)}\right) \tag{3}$$

where

$$x = 2[E^*]_T - [T]_0 + \sqrt{([T]_0 + K_E)^2 - 4([T]_0 - [E^*]_T)[E^*]_T} \tag{4}$$

and

$$x_0 = 2[E^*]_0 - [T]_0 + \sqrt{([T]_0 + K_E)^2 - 4[E^*]_0[E]_0} \tag{5}$$

The tunable parameters $K_M$ and $k_{cat}$ are now $K_E$ and $k_c$, the dissociation constant for unphosphorylated-phosphorylated CCA complex formation, and the first-order rate constant for the catalytic phosphorylation reaction, respectively. In our model, microtubules increase the rate of formation of $[E^*]_T$ by increasing CCA collision rates (increasing $k'_1$ and/or $k'_2$, which means decreasing $K_E$ and/or $K'_E$) through a reduction in dimensionality. An example plot of $[E^*]_T$ as a function of $t$ using Equation 3 is shown below. Here, $[E^*]_0 = 0.1$ $\mu$M, $[E_0] = 0.9$ $\mu$M, $k_c = 1$ min$^{-1}$ and $K_E$ is varied.

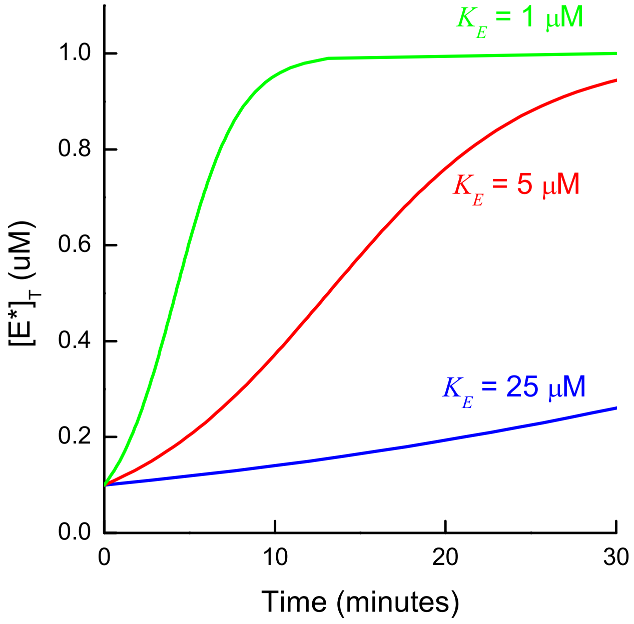


## Reduction in Dimensionality

From Berg and Purcell [2, 3], let $W(r)$ be the time it takes for a particle to encounter an absorbing sphere of radius $a$. A reflecting boundary exists at $r = b > a$. In general, $W$ is described by:

$$\nabla^2 W + \frac{1}{D_n} = 0 \tag{6}$$

where $D_n$ is the diffusion coefficient in n-dimensions. In 3-dimensions, the solution for $W(r)$ with the above boundary conditions ($W(r = a) = 0$ and $\nabla W(r = b) = 0$) is given by:

$$W(r) = \frac{\frac{2b^3}{a} - \frac{2b^3}{r} + a^2 - r^2}{6D_3} \tag{7}$$

Here, $D_3$ is the diffusion coefficient in 3-dimensions. We want the *mean* of this so-called "first passage time", or $\tau_{\mathrm{MFPT}}$. This is the average of $W(r)$ when the particle is placed at random inside the volume many times and is defined as:

$$\tau_{\mathrm{MFPT}} = \frac{1}{\pi(b^2 - a^2)} \int_a^b W(r)dr = \frac{b^6}{3D_3 a(b^3 - a^3)} \left(1 - \frac{9a}{5b} + \frac{a^3}{b^3} - \frac{a^6}{5b^6}\right)$$

$$\text{and when } b \gg a, \boxed{\tau_{\mathrm{MFPT}} \approx \frac{b^3}{3aD_3}} \tag{8}$$

Let the particle be a phosphorylated CCA molecule and the absorbing sphere an unphosphorylated CCA. If we assume $a \approx 2$ nm (radius of a typical globular protein) and the viscosity $\eta \approx 1$ cP, then from the Einstein-Stokes relation we can estimate $D_3 = \frac{k_B T}{6\pi\eta r} \approx 10\ \mu\mathrm{m}^2\ \mathrm{s}^{-1}$ at 25°C. Both the particle and the absorbing sphere undergo diffusion, however, which means that the relative diffusion coefficient is $D_3 = D_{\mathrm{particle}} + D_{\mathrm{absorbing\ sphere}}$. Assuming that phosphorylation does not affect diffusion coefficients, $D_{\mathrm{particle}} = D_{\mathrm{absorbing\ sphere}}$, and $D_3 \approx 20\ \mu\mathrm{m}^2\ \mathrm{s}^{-1}$.

In the 1-dimensional case, Equation 6 becomes:

$$\frac{d^2 W(x)}{dx^2} + \frac{1}{D_1} = 0 \tag{9}$$

where $D_1$ is the 1-dimensional diffusion coefficient (which we have measured for CCA using single-molecule TIRF). We imagine that a CCA molecule diffuses along the $x$-axis at $0 < x \leq b$. $x = 0$ is an absorbing point, and $x = b$ is a

reflecting boundary, as in the 3-dimensional case. The boundary conditions to solve Equation 9 are $W(x=0)=0$, and $\frac{dW(x=b)}{dx}=0$. The solution is:

$$W(x) = \frac{1}{2D_1}(2bx - x^2)$$

The mean first passage time for the 1D case is $\frac{1}{b-a}\int_a^b W(x)dx = \boxed{\tau_{\text{MFPT}} = \frac{b^2}{3D_1}}$.

We are interested in knowing if 1D diffusion produces smaller mean first passage times than in 3D. The equations for $\tau_{\text{MFPT}}$, however, both depend on $b$, the separation distance between the absorbing and reflective boundaries. We can solve for a value of $b$ for which $\tau_{\text{MFPT in 1D}} < \tau_{\text{MFPT in 3D}}$:

$$b > a\frac{D_3}{D_1} \tag{10}$$

which, using the above values for the diffusion coefficients and $a \approx 2$ nm, gives $b > 0.4$ $\mu$m. The dependency on the value of $b$ is clearly shown in Figure 3D of the main paper.

### Supplementary References


[1] Z. X. Wang and J. W. Wu. Autophosphorylation kinetics of protein kinases. *Biochem J*, 368:947–952, 2002.

[2] H. C. Berg and E. M. Purcell. Physics of chemoreception. *Biophys J*, 20(2):193–219, 1977.

[3] H. C. Berg. *Random Walks in Biology.* Princeton University Press, 1993.
